# Legislation

**Published:** 1895-04

**Authors:** 


Secretary.

---

### LEGISLATION.

### NATIONAL LEGISLATION.

### SALARIES AND EXPENSES, BUREAU OF ANIMAL INDUSTRY.

*Extract from Bill No.* 8927, *House of Representatives. United States Department of Agriculture.*

FOR carrying out the provisions of the Act of May 29, 1884, establishing the Bureau of Animal Industry, and of the Act of August 30, 1890, providing for an inspection of meats and animals, also the provisions of the Act of March 3, 1891, providing for the inspection of live cattle, hogs, and the carcasses and products thereof which are the subjects of interstate and foreign commerce, and for other purposes, the sum of $800,000; and the Secretary of Agriculture is hereby authorized to use any part of this sum he may deem necessary or expedient, and in such manner as he may think best, in the collection of information concerning livestock, dairy and other animal products, and to prevent the spread of pleuro-pneumonia, tuberculosis, sheep-scab, and other diseases of animals, and for this purpose to employ as many persons as he may deem necessary, including \$1000 additional temporary compensation to the Chief of the Bureau of Animal Industry, and to expend any part of this sum in the purchase and destruction of diseased or exposed animals, and the quarantine of the same whenever in his judgment it is essential to prevent the spread of pleuro-pneumonia, tuberculosis, or other diseases of animals from one State into another, and for printing and publishing such reports relating to animal industry as he may direct; and the Secretary is hereby authorized to rent a suitable building in the District of Columbia, at an annual rental of not exceeding \$1200, to be used as a laboratory for said Bureau of Animal Industry (23); *Provided*, That Sec. 2 of the Act entitled "An Act to provide for the inspection of live cattle, hogs, and the carcasses and products thereof which are the subjects of interstate commerce, and for other purposes," approved March 3, 1891, be amended to read as follows:

"SEC. 2. That the Secretary of Agriculture shall also cause to be made a careful inspection of all live cattle, the meat of which, fresh, salted, canned, packed, cured, or otherwise prepared, is intended for exportation to any foreign country, at such times and places, and in such manner as he may think proper, with a view to ascertain whether said cattle are free from disease, and their meat sound and wholesome, and may appoint inspectors who shall be authorized to give an official certificate clearly stating the condition in which such cattle and meat are found, and no clearance shall be given to any vessel having on board any fresh, salted, canned, corned, or packed beef, being the meat of cattle killed after the passage of this Act, for exportation to and sale in a foreign country from any port in the United States until the owner or shipper shall obtain from an inspector appointed under the provisions of this Act a certificate that said cattle were free from disease and that their meat is sound and wholesome."

(24.) Also that Sec. 4 of said Act be so amended as to read as follows:

"SEC. 4. That said examination shall be made in the manner provided by rules and regulations to be prescribed by the Secretary of Agriculture, and after said examination the carcasses and products of all cattle, sheep, and swine found to be free of disease and wholesome, sound, and fit for human food shall be marked, stamped, or labelled for identification as may be provided by said rules and regulations of the Secretary of Agriculture. Any person who shall forge, counterfeit, simulate, imitate, falsely represent, or use without authority, or knowingly and wrongfully alter, deface, or destroy any of the marks, stamps, or other devices provided for in the regulations of the Secretary of Agriculture, of any such carcasses or their products, or who shall forge, counterfeit, simulate, imitate, falsely represent, or use without authority, or knowingly and wrongfully alter, deface, or destroy any certificate or stamp provided in said regulations, shall be deemed guilty of a misdemeanor, and on conviction thereof shall be punished by a fine not exceeding $1000, or imprisonment not exceeding one year, or by both said punishments, in the discretion of the court."

(25.) The Secretary is hereby authorized to make such rules and regulations as he may decide to be necessary to prevent transportation from one State or Territory or the District of Columbia into any other State or Territory or the District of Columbia, or to any foreign country, of the condemned carcasses or parts of carcasses of cattle, sheep, and swine which have been inspected in accordance with the provisions of this Act. Any person, company, or corporation owning or operating any such slaughter-house, abattoir, or meat-curing, packing, or canning establishment, or any employé of the same, that shall wilfully violate any provision of this Act shall be deemed guilty of a misdemeanor, and on conviction thereof shall be punished for each offense by a fine not exceeding $1000, or imprisonment not exceeding one year, or by both said punishments, in the discretion of the court.

Quarantine stations for neat cattle: To establish and maintain quarantine stations, and to provide proper shelter for and care of neat cattle imported at such ports as may be deemed necessary, $12,000.

That whenever the Secretary of Agriculture shall certify to the (26) President of the United States what countries or parts of countries are free from contagious or infectious diseases of domestic animals, and that neat cattle and hides can be imported from such countries without danger to the domestic animals of the United States the (27) President of the United States may suspend the prohibition of the importation of neat cattle and hides in the manner provided by law. That the President of the United States be and is hereby authorized to cause correspondence and negotiation to be had, through the

Department of State or otherwise, with the authorities of the Kingdom of Great Britain, for the purpose of securing the abrogation or modification of the regulations now enforced by said authorities which require cattle imported into Great Britain from the United States of America to be slaughtered at the port of entry, and prohibiting the same from being carried alive to other places in said Kingdom.

That the Secretary of Agriculture shall determine and certify to the Secretary of the Treasury what are recognized breeds and pure bred animals, under the provisions of paragraph (28) three hundred and seventy-three of the (29) Tariff Act of (30) 1894.

---

### STATE LEGISLATION.

### Massachusetts.

*Synopsis of Bill.*—A bill has been introduced into the Legislature of Massachusetts to provide for the registration of veterinary physicians and surgeons.

Section 1 provides that said board be composed of five graduates from legally chartered veterinary college or colleges, and who shall have been in active practice for a period of ten years, and to provide for their term of office and any vacancies that may occur and for removal for cause of any member of said board.

Sec. 2 provides for the organization of the board in the election of chairman and secretary. The secretary shall give to the treasurer of the Commonwealth a bond in the penal sum of $2000 for the faithful discharge of his duties. Also, that three regular meetings shall be held in each year in February, June, and October.

Sec. 3 provides for the notification of all veterinary practitioners of the provisions of the act through newspaper channels. Graduates and non-graduates who have been in continuous practice for a period of three years immediately preceding the passage of the act shall be entitled to registration upon the payment of the fee of $1.

Sec. 4 provides for revoking certificates of registration of any one convicted of a crime in the course of professional business, and the cancellation of the registration and the payment of all fees into the treasury of the Commonwealth.

SEC. 5 provides for the compensation and expenses of the board.

SEC. 6 provides for the report of all registrations, moneys received and expended, the same to be open to inspection in the office of the Commonwealth. Also fixes the annual report to the Governor, together with a statement of the condition of veterinary science and report of the board's work during the year.

SEC. 7 provides that said board shall investigate the violations of the act, and place the same in the hands of the proper prosecuting officers.

SEC. 8 fixes January 1, 1896, as the day when all applicants for registration shall be filed. Applicants must be twenty-one years of age, of good moral character, and no person shall be entitled to register without examination after said day.

SEC. 9 provides for the character of examination.

SEC. 10 provides penalty for practice without registration.

SEC. 11 exempts commissioned officers of the United States service, or visiting physician or surgeon from another State, and does not prohibit gratuitous services.

SEC. 12. This act shall take effect upon its passage.

### DISTRICT OF COLUMBIA.

*Synopsis of Bill.*—The act to regulate the practice of veterinary medicine and surgery of the District of Columbia was not presented at the session of Congress just closed, owing to the fact that there was little chance of its passage at the late time of the preparation of the bill.

SECTION 1 of the bill calls for a commission to be known as "The District of Columbia Veterinary Medical Board," to consist of three commissioners, members in good standing of the Veterinary Medical Association of the District of Columbia, who shall be appointed by the Commissioners of the District of Columbia every three years, with power to adopt such laws and regulations as are necessary to carry into effect the provisions of the act.

SEC. 2 makes it unlawful for any person or persons to practice veterinary medicine or surgery in the District of Columbia without a diploma from a duly authorized college, and having passed a satisfactory examination before the commissioners.

SEC. 3 provides for the meetings.

SEC. 4 provides for the examination of diplomas as to their genuineness, and written or oral, theoretical and practical, examinations to be satisfactory to the board before the issuing of the license.

SEC. 5. All examinations of non-graduates shall be made by the board, and must take place within six months after the passage of the act.

SEC. 6 reads as follows: That any person shall be regarded as practicing veterinary medicine and surgery within the meaning of this act who shall append to their names the letters V.S., or D.V.S., or the words veterinary surgeon, veterinarian, or veterinary dentist, or who shall prescribe, advise or apply any drugs or medicines or other agents, or who shall perform any operation for the treatment, cure, or relief of any sick or diseased domestic animal, or who shall publicly profess to do any of these things.

SEC. 7 provides for the register of all practitioners qualified under the act, the same to be published at least once a year in two newspapers in the city of Washington.

SEC. 8 provides for a register of all those who are practicing under a license of the board, and that no non-graduate shall be allowed to register who has not been in practice for five years preceding the passage of the act.

SECS. 9 and 10 give power to reject applicants for registration whose examination papers and diplomas are not satisfactory, and that no persons shall practice after six months from the approval of the act without such examination and registration.

SEC. 11 provides penalties for violations of the act.

SEC. 12 provides that the board shall be a prosecutor in all cases of violation.

SEC. 13 provides that certificates of registration shall be filed with the health officers of the District of Columbia.

SEC. 14 provides that this act shall take effect immediately after its passage.

WE regret to learn that the efforts to secure a State Veterinarian in California have proved unavailing, though earnest work was done by the veterinarians for its accomplishment. We wish them better fortune next time.

The bill to regulate the practice of veterinary medicine and surgery pending before the Wisconsin State Legislature failed to become a law. Veterinarians must learn as individuals and as component parts of their professional organizations that one and all must work early and late to secure such recognition, and that no matter how earnest and efficient the officers may be the members must give them an unbroken column of support. Never was this fact more forcibly impressed upon the profession of any State than it was in Pennsylvania the past winter.

